# Interpreting the Effect of Stimulus Parameters on the Electrically Evoked Compound Action Potential and on Neural Health Estimates

**DOI:** 10.1007/s10162-020-00774-z

**Published:** 2020-10-27

**Authors:** Tim Brochier, Colette M. McKay, Robert P. Carlyon

**Affiliations:** 1 Cambridge Hearing Group, MRC Cognition and Brain Sciences Unit, University of Cambridge, 15 Chaucer Road, Cambridge, CB2 7EF UK; 2 Bionics Institute, 384-388 Albert Street, East Melbourne, VIC 3002 Australia

**Keywords:** cochlear implants, neural health, neural survival, ECAP, electrophysiology, inter-phase gap

## Abstract

Variations in the condition of the neural population along the length of the cochlea can degrade the spectral and temporal representation of sounds conveyed by CIs, thereby limiting speech perception. One measurement that has been proposed as an estimate of neural survival (the number of remaining functional neurons) or neural health (the health of those remaining neurons) is the effect of stimulation parameters, such as the interphase gap (IPG), on the amplitude growth function (AGF) of the electrically evoked compound action potential (ECAP). The extent to which such measures reflect neural factors, rather than non-neural factors (e.g. electrode orientation, electrode-modiolus distance, and impedance), depends crucially upon how the AGF data are analysed. However, there is currently no consensus in the literature for the correct method to interpret changes in the ECAP AGF due to changes in stimulation parameters. We present a simple theoretical model for the effect of IPG on ECAP AGFs, along with a re-analysis of both animal and human data that measured the IPG effect. Both the theoretical model and the re-analysis of the animal data suggest that the IPG effect on ECAP AGF slope (IPG slope effect), measured using either a linear or logarithmic input-output scale, does not successfully control for the effects of non-neural factors. Both the model and the data suggest that the appropriate method to estimate neural health is by measuring the IPG offset effect, defined as the dB offset between the linear portions of ECAP AGFs for two stimuli differing only in IPG.

## Introduction

Cochlear implants (CI) provide a sense of hearing to people with sensorineural hearing loss by directly stimulating auditory nerve fibres via an implanted array of electrodes. Variations in neural health along the length of the cochlea can degrade the spectral and temporal representation of sounds conveyed by CIs, thereby limiting speech perception.

In order to explain and potentially remedy cases of poor speech perception by CI users, several methods to estimate neural health have been proposed. Measurements derived from the electrically evoked compound action potential (ECAP) have been correlated with SGN density in animals (Shepherd and Javel 1997; Prado-Guitierrez et al. 2006; Ramekers et al. 2014) and with speech perception in humans (Kim et al. 2010). ECAP measures are objective and time-efficient and can be measured with the device itself, without the need for additional hardware. These factors make ECAPs a viable clinical method for estimating neural health. The aim of this paper is to review ECAP-based methods of estimating neural survival and neural health, to identify the most informative measures, and to provide a tool that will assist researchers in evaluating their ECAP data.

Throughout this paper, the terms “neural survival” and “neural health” will be used to describe two separate, distinct features of the neural population. Neural survival refers to the *number* of remaining neurons, while neural health refers to the *health* of those remaining neurons, which may be affected by the physiological properties of the peripheral processes, the cell body, and the central axon (Brochier et al. 2020). In most pathologies and in deafened animals, neural health and neural survival covary, the neurons progressively degenerate, thereby reducing the overall number of neurons. However, neural health and neural survival may have different effects on ECAP measurements, and will be represented as separate factors in the model presented here.

Several ECAP features have been positively correlated with spiral ganglion neuron (SGN) density in animals. These features have included maximum ECAP amplitude, the slope of the ECAP amplitude growth function (AGF), and the offset between stimuli of different IPGs needed to elicit an ECAP of the same amplitude (Prado-Guitierrez et al. 2006; Ramekers et al. 2014). In those studies, comparisons were made between groups that were deafened for different amounts of time, and the short-termed deafened group generally had higher SGN density, higher ECAP amplitudes, steeper ECAP AGF slopes, and larger IPG offsets. To translate this research towards a clinical benefit for CI users, several authors have extended the use of these methods beyond group comparisons in animals to within-subject comparisons of different CI electrodes in human implant users. Estimations of neural health and neural survival along the length of the cochlea may help to optimize processing strategies, by informing the deactivation of electrodes in unhealthy, sparse neural regions (Garadat et al. 2013; Goehring et al. 2019) or the application of focussed stimulation in healthy, dense neural regions (Bierer 2010).

Despite the across-subject correlation between animal SGN survival and ECAP parameters, ECAPs may be affected by non-neural factors including the electrode-modiolus distance (EMD) and/or the impedances of both the stimulating and recording electrodes, as well as their orientation relative to the stimulated neurons. These additional parameters may be more important in studies with humans, who, compared with the animals in physiological studies, have larger cochleae, electrode arrays that vary in distance from the modiolus, and a longer duration of deafness (which may lead to fibrous tissue growth around parts of the electrode array). Consequently, channel-to-channel variation in absolute measures such as ECAP amplitude or ECAP AGF slope might reflect factors unrelated to neural survival or neural health (Schvartz-Leyzac and Pfingst 2016). The influence of these factors could lead to inaccurate estimates of neural survival or neural health. For example, an electrode that stimulates a dense neural population might have a low maximum ECAP if the recording electrode were relatively far away from the modiolus.

To overcome the influence of non-neural factors on absolute ECAP measures, differential ECAP measures have been proposed. Two studies on guinea pigs provided evidence that the effect of increasing the interphase gap (IPG) on ECAP responses was positively correlated with SGN density. In one of these, Prado-Guitierrez et al. (2006) measured the difference in current level (dB) required to elicit an equal normalized amplitude ECAP at an IPG of 8 μs compared with an IPG of 58 μs. This IPG offset effect was positively correlated with SGN cell density in guinea pigs tested after 1, 4, or 12 weeks of deafness. Ramekers et al. (2014) replicated the results of Prado-Gutierrez et al. (2006), showing that the IPG offset effect was positively correlated with SGN density in guinea pigs. Additionally, Ramekers et al. (2014) measured the slope of the ECAP AGF on linear input-output coordinates for IPGs of 2.1 and 30 μs and found that the difference between these slopes was positively correlated with SGN density in implanted normal-hearing, 2-week deafened, and 6-week deafened guinea pigs. Throughout this article, the measure of the difference between ECAP AGF slopes for different IPGs will be referred to as the IPG slope effect, and the horizontal shift of ECAP AGFs for different IPGs will be referred to as the IPG offset effect.

The potential of difference measures to partial out non-neural effects, which are assumed to be the same across conditions, has led to measures such as the IPG slope effect and the IPG offset effect being commonly used to estimate local neural health along the array in humans (Kim et al. 2010; McKay and Smale 2017; Hughes et al. 2018; Schvartz-Leyzac and Pfingst 2018). Here we argue that the extent to which non-neural influences are removed depends crucially on how the data are analysed. In particular, we argue that comparing the IPG slope effect, either between subjects or between electrodes in the same subject, can lead to significant differences that are (sometimes trivially) due to non-neural factors. Similar conclusions apply to other stimulus manipulations, such as phase duration or stimulus polarity, but we focus on the effect of IPG to illustrate the general principle because animal physiological data are available to help elucidate the relation to neural health and because of the emerging interest in the effect of IPG as a clinical tool.

In the following sections, the IPG slope effect and the IPG offset effect will be examined using a simple theoretical model, which we then apply to animal data kindly provided by Prado-Guitierrez et al. (2006), and to human CI user data collected by McKay and Smale (2017). Specifically, we will assess the use of linear and logarithmic scales, normalization, linear and sigmoidal fitting functions, and slope and amplitude comparisons, with the aim of identifying the most informative measures. We will then propose a standardized IPG offset effect measurement (similar to Prado-Gutierrez et al. (2006)) that is intuitive, accurate, and easy to calculate.

## Methods

The simple theoretical model was implemented in MATLAB (MathWorks, version R2019b), and statistical analysis of the animal data (Prado-Guitierrez et al. 2006) and human CI user data (McKay and Smale 2017) was performed in IBM SPSS Statistics (version 26). Code for a MATLAB GUI that calculates the IPG offset effect has been added to our public git repository (https://github.com/tjbrochier/ECAP-AGF-Methods).

### A Simple Theoretical Model of ECAP Amplitude Growth

#### Linear Model

Here a simple theoretical model is used to demonstrate features that can be extracted from the ECAP AGF and to illustrate how those features are affected by different analysis techniques. It is independent of the particular ECAP measurement method, such as the forward masking vs alternating polarity methods. To illustrate the general principles behind our argument we take a very simple case in which the size of the ECAP is determined by the input current (*I*) multiplied by the product of stimulating factors, raised to the power *k*, and then multiplied by the product of recording factors:

1 $$V=r\bullet n\bullet {\left(s\bullet g\bullet I\ \right)}^k$$

where ***r*** reflects characteristics of the ***r***ecording electrode, such as its lateral position and impedance, ***n*** reflects the number of the ***n***eurons near the stimulating electrode, ***s*** reflects characteristics of the ***s***timulation electrode, and ***g*** is a ***g***ain factor determined by stimulus characteristics such as IPG, phase duration, waveform shape, and polarity. The exponent ***k*** relates the effective current at the neurons to neural activity, which is influenced by the fibre threshold distribution of the neural population (Miller et al. 1999). Our choice of a power function was based upon measurements of auditory nerve fibres in cats (Sachs and Abbas 1974; Shepherd and Javel 1999; Miller et al. 1999) and psychophysical studies of loudness growth with electrical stimulation in humans (Zeng and Shannon 1992; McKay and McDermott 1998; Chatterjee et al. 2000; McKay et al. 2001, 2003). The stimulating factors ***s*** and ***g*** are multiplied by current *I* and raised to the power of *k* because they modify the effective current at the neurons (*I*) before that current is converted to neural excitation (which can be represented by a power function characterized by ***k***). In contrast, the “response factors” ***r*** and ***n*** have no effect on the input current, and so they are not raised to the power of ***k***.

It is worth noting that an important assumption of the model is that the parameters that affect the ECAP do so in a multiplicative manner. This assumption is based on the effect of, say, the electrode-modiolus distance of the ***s***timulating electrode producing a proportional change in the current reaching each neuron, with threshold current increasing approximately with the square of the distance from the stimulating electrode (BeMent and Ranck 1969a, b; Ranck 1975; Rattay 1989; Abbas and Miller 2013). Similarly, increasing the EMD of the ***r***ecording electrode will decrease the ECAP by a certain proportion, regardless of the size of the voltage at the neurons (that proportion will also be independent of the exponent ***k***). Biophysical models (Shepherd and Javel 1999; Abbas and Miller 2013) account for the effects of stimulus characteristics such as IPG (the ***g*** factor in our model) in terms of integration of charge at the level of the cell membrane, such that a given change in IPG corresponds to a proportional decrease in the current needed to produce a criterion depolarisation.

We start with the case where *k* = 1, both for simplicity and because this is required for approaches that measure the slope of the AGF on linear-linear coordinates. It can be seen that the slope, *dV/dI*, will depend on all four factors (*r*, *s*, *n*, and *g*), and the difference between slopes measured with different IPGs (different *g* factors) can be expressed as:

2 $$\frac{d{V}_1}{dI}-\frac{d{V}_2}{dI}=r\ s\ n\kern0.5em \left({g}_1-{g}_2\right)$$

**Fig. 1:**
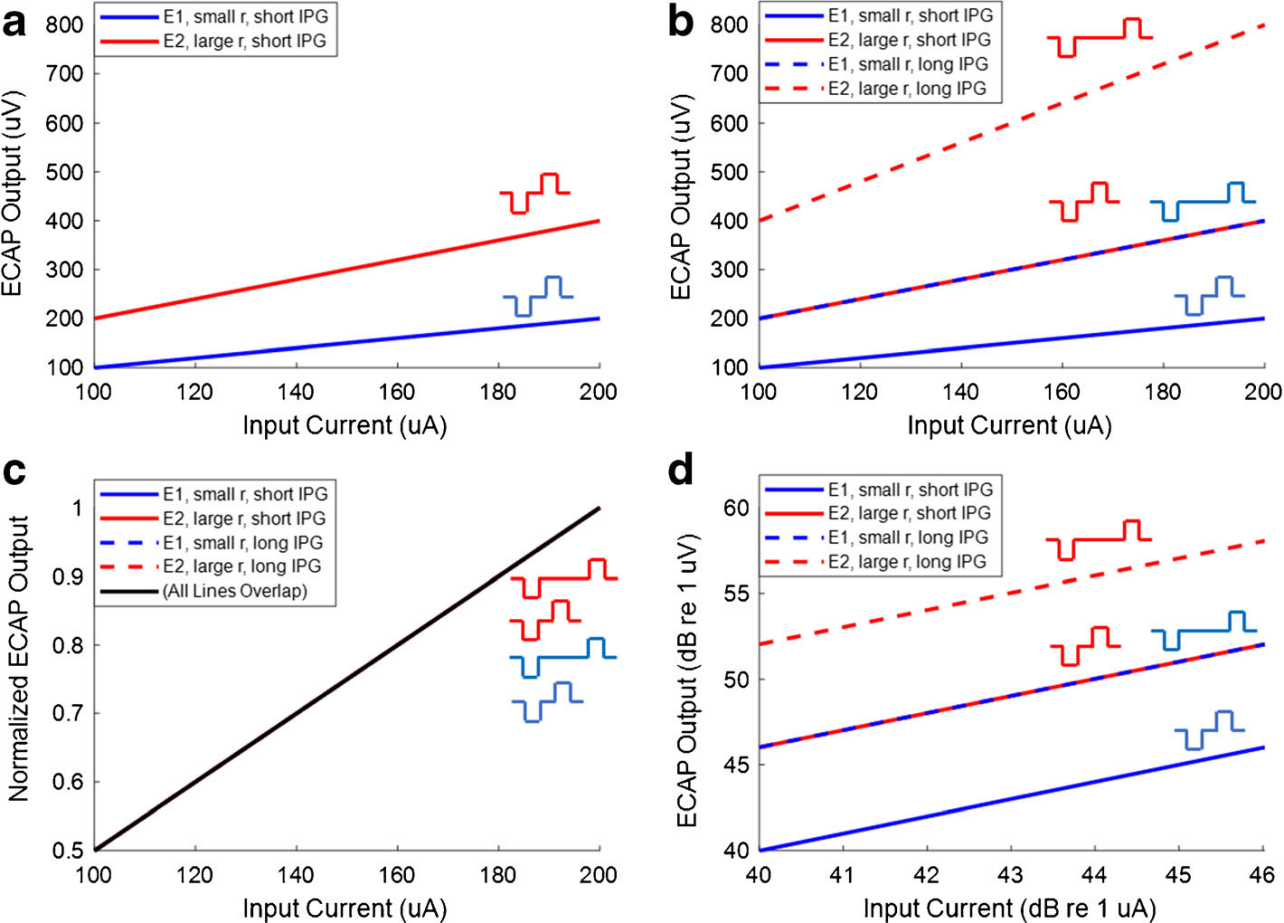
The four panels demonstrate the simple linear model of ECAP AGFs, and the effect of different scaling methods on the calculation of the IPG effect on slope. In **panel A**, ECAP AGFs are shown for electrode 1 (E1, blue) and electrode 2 (E2, red) on linear input and linear output coordinates. E1 and E2 differ by a factor of two in **r** (recording electrode characteristics), resulting in a steeper AGF slope for E2 compared to E1 but have equal neural health and neural survival. In **panel B** (also on a linear input-linear output scale), the dotted lines represent the ECAP AGFs of E1 and E2 after the IPG has been increased, resulting in a doubled ECAP amplitude for a given input current. Note that the IPG effect, calculated as the difference between linear slopes, will be larger for E2 than E1. In **panel C**, each of the ECAP AGFs in panel B has been normalized to their maximum ECAP output, resulting in completely overlapping ECAP AGFs. In **panel D**, logarithmic input and output coordinates are used, and the effect of IPG calculated as a difference between slopes shows that the effect of IPG is identical for E1 and E2 (zero effect in both cases)

Hence if we measure AGFs on two electrodes that differ by a factor of two in *r*, but are otherwise identical (including having equal neural health and neural survival), the slope of the AGF (in linear-linear units) where *r* is larger (e.g. recording electrode is closer to the modiolus) will be twice that of the electrode where *r* is smaller, as illustrated by the red and blue solid lines in Fig. 1a. Now assume that we change a stimulus parameter, such as the IPG, so as to produce a doubling in *g*, as shown by the dashed lines in Fig. 1b. It can be seen that the linear-linear slope of the AGF increases from 1 to 2 in one case (a difference of 1) but from 2 to 4 in the other (a difference of 2). Hence if one measures the difference in linear slope, then the “IPG slope effect” (measured as a difference in linear slopes) can double simply due to a difference in *r—*that is, in the characteristics of the recording electrode. The same will occur for a difference in the stimulating electrode characteristics, *s*. In both cases the linear-linear slope would differ between the two electrodes, but it would be incorrect to conclude that this IPG slope effect reflected a difference in neural survival. Note that, using linear-linear plots, the measured IPG slope effect would also depend on the number of remaining neurons, *n.* However the important point is that it is not possible to distinguish between an effect of *n* and effects of *r* or of *s*. Note also that it *is* possible (and, indeed likely) that the effect of a given IPG will depend on the biophysical properties of the nerve membrane that are stimulated (neural health), and that these properties might covary with neural survival (for example with the number of remaining peripheral processes). If so then *g* would covary with neural survival, and so would the IPG slope effect when measured as the difference between linear slopes. Again, however, it would not be possible to distinguish this effect from the effects of *r* or of *s.*

In a further effort to eliminate the effects of non-neural factors, several researchers (Kim et al. 2010; Hughes et al. 2018) have normalized the AGF to the maximum ECAP value obtained, as illustrated in Fig. 1c, again with linear coordinates for the abscissa (current) and ordinate (ECAP). Two points are worth making here. First, although the normalization controls for the effects of *r*, by scaling all ECAP amplitude values to the same range, it may not control for the effects of *s.* This is because, at least in human experiments, the (linear) range of stimulus levels used will be decreased whenever *s* is high, for example, because the stimulating electrode is closest to the modiolus. This will in turn decrease the range of values on the abscissa on linear coordinates (as all input currents are reduced by the same factor), thereby increasing the slope measured on linear coordinates, despite the normalization of the ECAP AGF. Because this effect is multiplicative, it will once again increase not only the slope but also the difference in slopes between two AGFs that differ in *g* (e.g., in IPG). Therefore, the IPG slope effect will be influenced by non-neural differences in ***s***, even if the output ECAP AGF is normalized. Second, even though the normalization of the ECAP AGF can control for some of the effects of using a linear ordinate, its effect will nevertheless depend on how it is applied. For example, if one normalizes all responses for a given subject to the maximum observed for all electrodes tested for that subject, then comparisons *between* electrodes will still be subject to the problem above, because the same scaling factor is used in all cases.

Another approach that has been adopted is to plot the stimulus current on a logarithmic scale and the ECAP output on a linear scale. McKay (2012) has convincingly argued that a dB scale is more appropriate than a linear scale when interpreting differences in current in psychophysical masking data, and the same principle applies for differences in the stimulus current in electrophysiological measurements. This is because the effect of, say, the electrode-modiolus distance of the stimulating electrode will produce a proportional rather than a linear change in the current reaching each neuron. However, the use of a linear scale for the ECAP output can still complicate the interpretation of, for example, effects of manipulating the IPG. If we let M = *logI*, then V = *r.s.n.g.* e^*M*^, and the slope *dV/dM* = *r.s.n.g.* e^*M*^. In other words, the slope increases with *M* and, for a given *M*, is proportional to *r.s.n.g.* This means that a change in *g* will have a proportional change on the slope, and so the linear slope change produced by, for example, a change in IPG, will be greater for an electrode with a large *r* than for one with a small *r.*

**Fig. 2:**
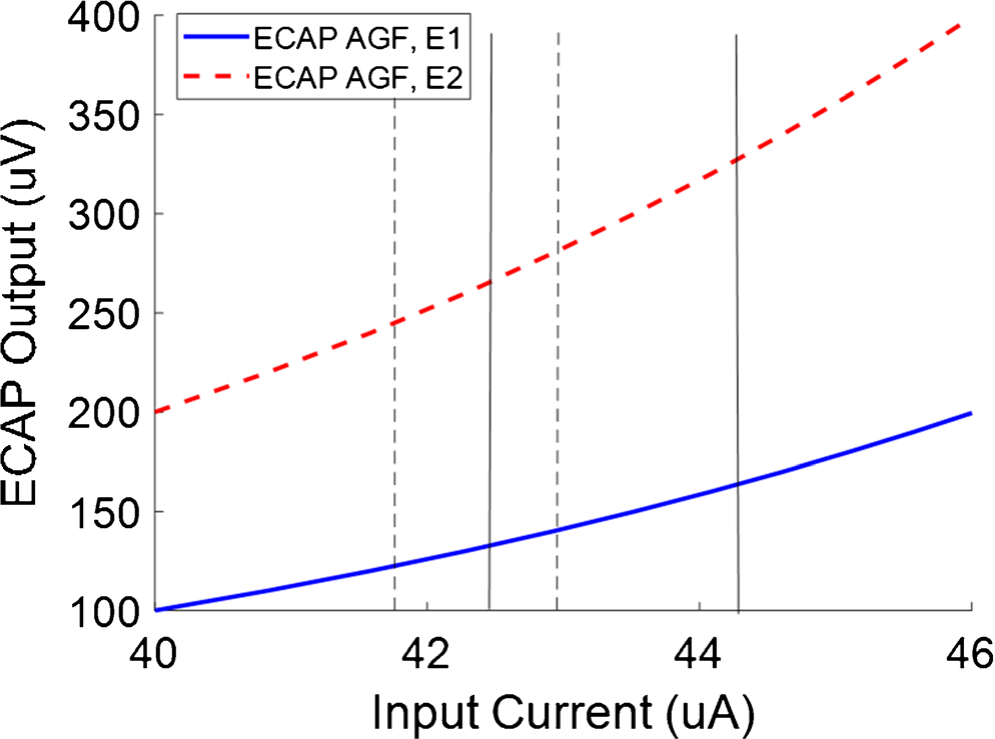
The curves above represent ECAP AGFs for two electrodes, E1 and E2, where the ECAP output is twice as large for E2. If the ECAP output is larger for E2 than E1 because of differences in recording electrode characteristics, the AGF will be measured over the same input current range for the two electrodes. If the ECAP output is larger for E2 than E1 because of differences in stimulating electrode characteristics, the AGF will be measured over different input current ranges for the two electrodes. For example, E1 may be measured using the stimulus range indicated by the solid vertical lines, whereas E2 may be measured over the range between the dashed lines. This will reduce the difference between the measured slopes of the two functions

Because the slope also changes with the range of input currents, the effects of differences between electrodes or subjects on the AGF slope depend on whether or not those differences cause the experimenter to change the range of input currents over which the slope is measured. Consider the example shown in Fig. 2, and assume that the solid and dashed curves show the AGFs for two functions that arise only from a difference in *r.* As noted above, when the slope is measured over the same range of input currents (shown by the solid vertical lines), it will be steeper for function E2, where *r* is larger. Changing *s* by the same factor will produce the same result. However, because a change in the stimulating electrode characteristics are also likely to affect loudness, then, at least in human experiments, the experimenter is likely to reduce the range of currents used for function E2. As shown by the dashed vertical lines, this will cause the function to be measured over a lower range of input currents, so that the effect of *s* on the measured slope is reduced or eliminated. Because the effect of *g* on the slope depends on the slope’s original value, this means that, in practice, the effect of IPG on slope will be measured as greater for a change in *r* than for an equivalent change in *s*.

An approach that eliminates the effects of *r* and *s* on the IPG effect is to plot the data on log-log co-ordinates (Fig. 1d):

3 $$\log V=\log r+\log n+k\left(\log s+\log g+\log I\right)$$

In this case, all factors have an additive effect and, under the null hypothesis that the IPG effect is independent of neural survival, all AGFs are offset by a constant value that depends on *r*, *s*, *n*, and *g*. However, a feature of this method is that there is no scope for neural survival or neural health to directly influence the slope of the AGF on log-log coordinates, *d(*log *V)/d(*log *I)*, which is equal to *k*. The difference or ratio between slopes measured with different pulse parameters (with assumed different *g* factors) will tend towards 0 and 1, respectively.

Thus, the use of the difference between AGF slopes to estimate neural survival or neural health is problematic using either linear or logarithmic input-output scales. With linear input-output scales, calculating the difference between AGF slopes does not remove the non-neural effects of ***r*** and ***s***. With logarithmic input-output scales, the AGF slope for all pulse parameters would be equal to ***k***, and the difference between slopes would be equal to 0. That is, the effects of ***r*** and ***s*** are removed but so are the effects of ***n*** and ***g***.

We suggest that a better method is to use the horizontal offset between AGFs with different pulse parameters (i.e. the “IPG offset”). The IPG offset can be represented by the difference between current levels *I*_*1*_ and *I*_2_, which generate equal ECAP outputs *V = V*_*1*_ = *V*_*2*_, with g factors *g*_*1*_ and *g*_*2*_:

$$ \log {I}_1-\log {I}_2=\kern0.5em \left(\frac{\log {V}_1-\log r-\log n}{k}-\log s-\log {g}_1\right)-\left(\ \frac{\log {V}_2-\log r-\log n}{k}-\log s-\log {g}_2\ \right) $$

4 $$ \log {I}_1-\log {I}_2=\log {g}_2-\log {g}_1\kern0.75em $$

As noted above, it may be that for a given change in IPG, the change in *g* will depend on the biophysical characteristics of the part of the membrane that is being stimulated and that this will vary with neural health. If so, then differences in neural health will be reflected as differences in the amount by which the IPG shifts the AGF horizontally (the IPG offset). The crucial difference between this approach and one that measures the difference between linear slopes is that non-neural factors such as ***r*** and ***s*** have no effect (as long as the IPG offset effect is quantified on a logarithmic scale). While neural survival (***n***) also has no effect, the effect of ***g*** is isolated and will reflect neural health. Based on our re-analysis of some animal data in “Methods” section, we will argue that the IPG offset effect, rather than the IPG slope effect, is indeed the most appropriate way to gauge neural health.

#### Limitations and Simplifying Assumptions of the Model

**Fig. 3:**
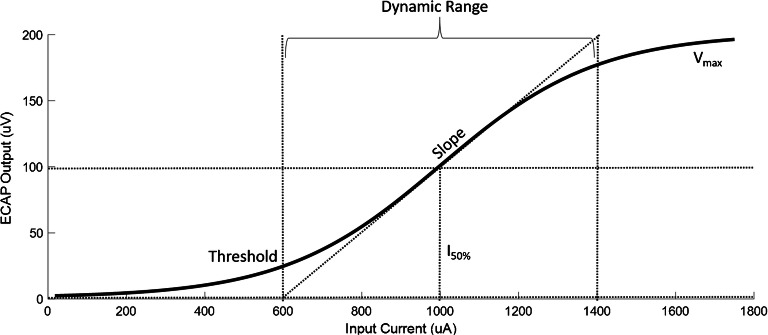
Diagram of sigmoid function representing a typical ECAP AGF and the features that can be extracted from it

The simple linear model described above is helpful for teasing apart the various factors that might influence the ECAP AGF and how this might be modified by changes in stimulus parameters. However, it makes several simplifying assumptions that should be discussed before proceeding with a re-analysis of the animal data. First, the simple model assumes that the AGF is linear over its entire range, which is not generally true. Rather, the slope is generally shallow at low input levels, both with linear and logarithmic input scales, and, at least in animal studies, reaches an asymptote at high levels. A simple fitting function that has been applied to the ECAP recorded by stimulating one electrode and recording from another is a sigmoid (Fig. 3; Ramekers et al. 2014):

Features extracted from the sigmoidal AGF are threshold, *Vmax*, slope, and *I*_*50%*_, all of which may be affected by the ***r***, ***s***, ***n***, and ***g*** factors mentioned above. When calculating the IPG slope effect (Ramekers et al. 2014) or the IPG offset effect (Prado-Guitierrez et al. 2006; Ramekers et al. 2014), the shallow portion of the AGF at low levels and the saturated portion of the AGF at high levels are disregarded, and the region of interest is restricted to the linear portion of the AGF. Therefore, the same reasoning described in the simple linear model applies.

Another simplification that the model makes is the use of the factor ***n*** to represent the total number of neurons, which implies that the contribution to the total ECAP amplitude for each neuron is characterized by the exact same ***r***, ***s***, ***g***, and ***k*** factors. In reality, a unique ***r***, ***s***, ***g***, and ***k*** would apply to each neuron, and the equation could be represented as the following:

5 $$V=\sum \limits_{i=1}^n{r}_i\bullet {\left({s}_i\bullet {g}_i\bullet I\ \right)}^{k_i}\kern0.5em$$

In this equation, ***n*** still represents the number of neurons. We implicitly assume that the ***k*** factor applies to each neuron separately and that the total neural response is calculated by summing the responses after that application of ***k****.* If each neuron is identical, then Eq. (5) is equivalent to Eq. (1). More generally, if the previous analysis is applied to the Eq. 5, with AGF slopes measured on linear coordinates, the results from the simplified model still hold; IPG slope effect is prone to influence by non-neural factors:

6 $$\frac{d{V}_1}{dI}-\frac{d{V}_2}{dI}=\sum \limits_{i=1}^n{r}_i\ {s}_i\ {k}_i\kern0.5em \left({g}_{1_i}{\left({s}_i\ {g}_{1_i}\ I\right)}^{k_i-1}-{g}_{2_i}{\left({s}_i\ {g}_{2_i}\ I\right)}^{k_i-1}\right)$$

Conversely, the IPG offset effect is solely dependent upon the ***g*** factor, which will depend upon the biophysical properties of the part of the membrane that is being stimulated:

7 $$\log {I}_1-\log {I}_2=\sum \limits_{i=1}^n\log {g}_{2_i}-\log {g}_{1_i}\kern0.5em$$

Even this “weighted sum” model assumes the unitary response concept for the ECAP, where the ECAP represents the summed total of individual neural responses. Westen et al. (2011) suggest that the unitary response concept is an oversimplification, and show in both guinea pigs and in a computational model that ECAP amplitudes begin to drop at sufficiently high levels due to overstimulation of the nerve fibres. However, the region of interest for both the IPG offset effect and the IPG slope effect is the monotonic, linear portion of the ECAP AGF, at levels below where the ECAP AGF saturates. We therefore believe that the simplifying assumption that more neural activation leads to higher ECAPs is appropriate, at least within the context of our simple model.

### Animal Data Analysis

Prado-Guitierrez et al. (2006) provided data that measured the effect of IPG on ECAP AGFs in 15 guinea pigs that were separated into three groups of five, deafened for either 1, 4, or 12 weeks before cochlear implantation. ECAP AGFs were measured with IPGs of 8 μs and 58 μs, and SGN fibres were counted to calculate overall SGN density. Here we re-analyse these data so as to evaluate whether the effect of IPG slope effect, when defined using relative rather than absolute scales, correlates with SGN density. We note here that because data from deafened animals are less influenced by non-neural factors than data from humans, neural survival and neural health covary and are both related to SGN density (Wise et al. 2017; Ramekers et al. 2020). The IPG offset effect (offset of log current for equal ECAP amplitude) on *I*_50%_ will also be evaluated for the Prado-Guitierrez et al. (2006) data.

**Fig. 4:**
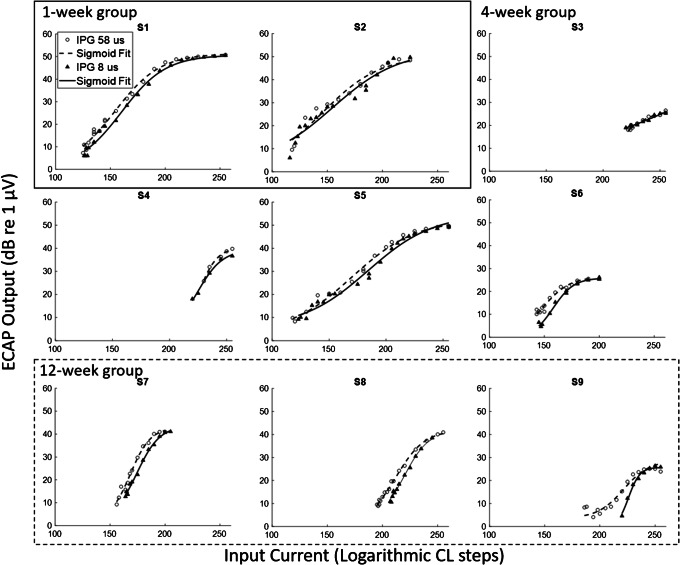
ECAP AGFs for 9 guinea pigs from the Prado-Guitierrez et al. (2006) study. The long IPG of 58 μs is represented by the open circles, and the short IPG of 8 μs is represented by the filled triangles. S1 and S2 were deafened for 1 week, S3–S6 were deafened for 4 weeks, and S7–S9 were deafened for 12 weeks

The ECAP AGFs are shown in Fig. 4 on log-log scales. S1 and S2 were from the 1-week deafened group, S3–S6 were from the 4-week deafened group, and S7–S9 were from the 12-week deafened group. As predicted by the simple linear model, the ECAP AGF slope on log-log coordinates did not correlate with SGN density (*R* = 0.034, *p* = 0.931) or with the IPG (*R* = 0.032, *p* = 0.905).

**Fig. 5:**
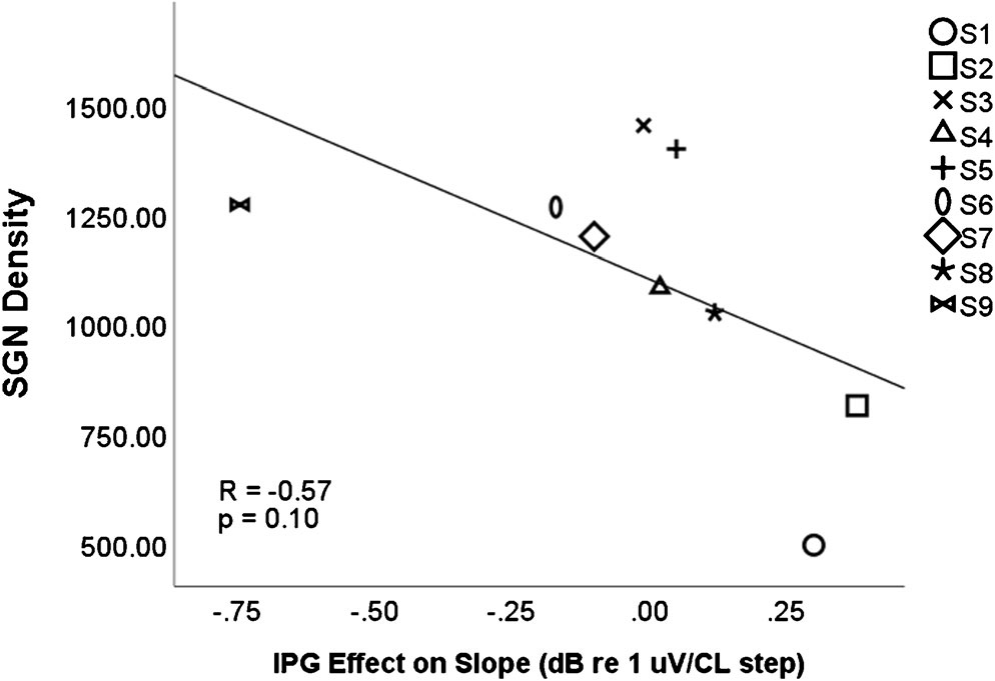
No correlation was found between SGN density and IPG effect calculated as a difference between slopes on a logarithmic scale

When we calculate the IPG slope effect using the difference between slopes calculated on a logarithmic input scale (in Cochlear CL steps) and a logarithmic output scale (in dB re 1 uV), there is no positive correlation across animals between SGN density and the IPG slope effect (*r* = − 0.57, *p* = 0.10), as shown in Fig. 5. No significant effect of IPG on slope was found (*R* = 0.05, *p* = 0.874, not shown), consistent with the predictions of the model in the previous section.

**Fig. 6:**
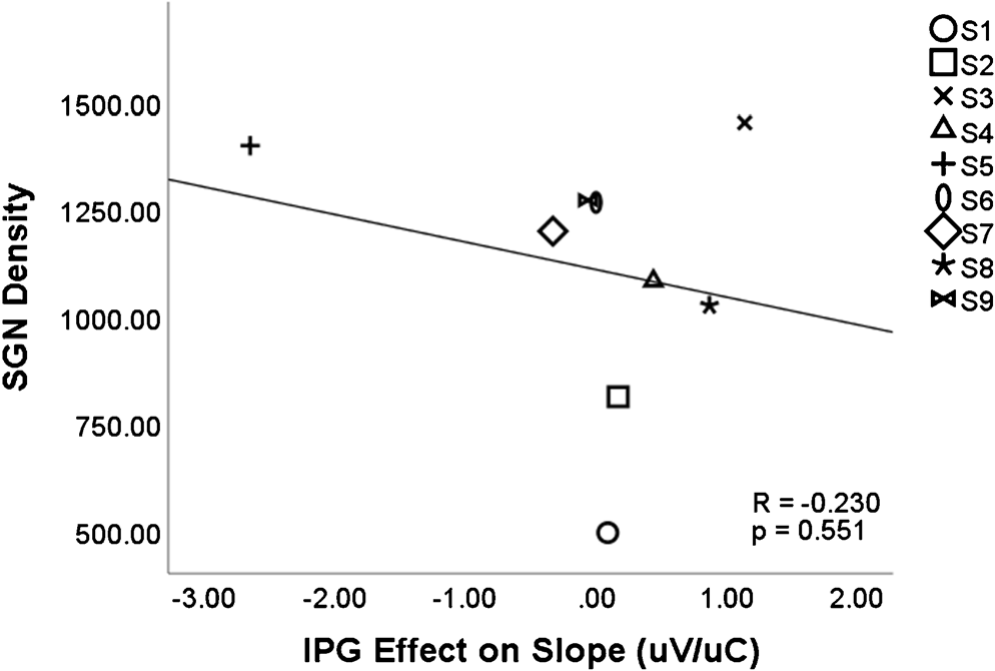
No correlation was found between SGN density and IPG effect calculated as a difference between slopes on a linear scale

Even when the IPG slope effect is measured using the difference between slopes calculated on a linear input and linear output scale, in the same way as Ramekers et al. (2014), there is no positive correlation between SGN density and IPG slope effect (*r* = − 0.23, *p* = 0.551), shown in Fig. 6. Because the IPG slope effect measured as the difference between linear slopes was not normally distributed across subjects, a Spearman nonparametric correlation was also performed, finding no significant relationship between the IPG slope effect and SGN density (*r* = − 0.200, *p* = 0.606).

The analyses of Prado-Guiterrez et al.’s data, described above, are consistent with our simple model but differ from the results of Ramekers et al. (2014), who found that the difference between AGF slopes on linear coordinates did correlate with SGN density. Some factors differed between the Prado-Guitierrez et al. (2006) and the Ramekers et al. (2014) data, which may have partly contributed to the difference in results. Prado-Guitierrez et al. (2006) used a long phase duration of 104 μs compared with the phase durations of 20–50 μs used by Ramekers et al. (2014). Ramekers et al. (2014) showed a reduction in the IPG slope effect (on a linear input-linear output scale) at larger phase durations. In addition, due to the artefact caused by the longer phase duration, Prado-Guitierrez et al. (2006) measured the P_2_–N_2_ amplitude in the ECAP response, rather than the N_1_–P_2_ amplitude. Even considering these differences, if the IPG slope effect was significantly positively correlated with SGN density at short phase durations, it would be surprising if the direction of the trend switched at longer phase durations. The lack of correlations in this data suggests that the IPG slope effect is not a robust way to predict SGN density.

**Fig. 7:**
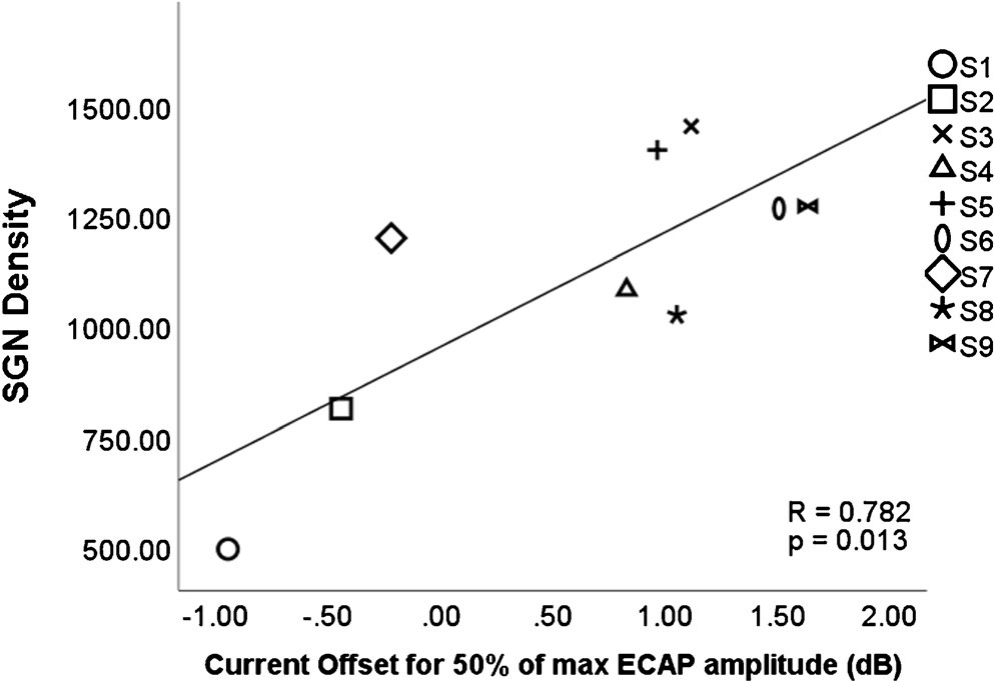
A significant positive correlation was found between SGN density and IPG effect calculated as a current offset between I_50%_ values between the different IPGs

In contrast, the positive correlation between IPG offset effect and SGN density was significant in both the Ramekers et al. (2014) data (*r* = 0.67, *p* < 0.05) and the Prado-Guitierrez et al. (2006) data (*r* = 0.782, *p* = 0.013, Fig. 7). Hence, unlike the case with the IPG slope effect, we do have evidence that the IPG offset effect, measured in this way, is a valid estimate of SGN density. Based on our theoretical model, we emphasize that this correlation is likely to be driven by neural health, not neural survival.

### Human Data Analysis

A re-analysis of ECAP AGF data provided by McKay and Smale (2017) shows again how the analysis method affects the interpretation of the IPG slope effect. In this data set, ECAP AGFs were measured for 10 CI users, with four combinations of IPGs (8 and 40 μs) and phase durations (25 and 40 μs). Up to 8 electrodes were measured across the array for each participant, and 52 electrodes out of the 80 that were tested had measurable ECAP AGFs in each of the conditions.

**Fig. 8:**
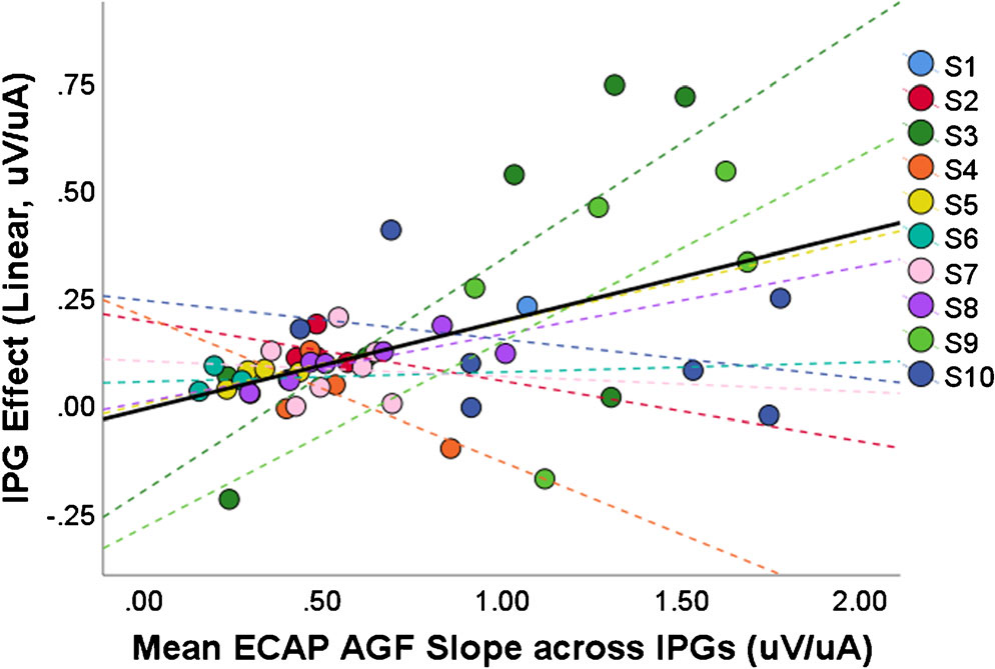
A significant positive correlation was found between the IPG effect measured as a difference between slopes on a linear scale and the mean ECAP AGF slope across IPGs

A one-way repeated measures ANOVA, with subject as a random factor, showed that IPG had a significant effect on ECAP AGF slope when calculated on a linear input and output scale (*p* = 0.041), with higher AGF slopes for the larger IPG. When the slope was calculated on a logarithmic input and output scale, the ECAP AGF slopes for the different IPGs were not significantly different (*p* = 0.766). In other words, the effect of IPG on the slopes can be simply interpreted as a constant change in gain applied to the ECAP. According to that model, any factors that increase the overall slope of the AGF will also increase the IPG slope effect, when measured as the difference in slopes measured on linear input and output scales. Consistent with this model, the IPG slope effect, when measured as the difference in linear slopes (μV/ μA), was correlated with the mean ECAP AGF slope (μV/ μA) across the two IPGs (*R* = 0.492, *p* = 0.012, Fig. 8). This correlation likely arises from the fact that for steeper overall ECAP AGFs, a given proportional change will necessarily lead to a larger IPG slope effect, if measured as a difference on a linear scale. It should not be interpreted as an indication that the linear IPG slope effect and the absolute linear slope are independently good estimates for neural health but rather that the two measures are mathematically related. Importantly, both measures are prone to influence by non-neural factors.

**Fig. 9:**
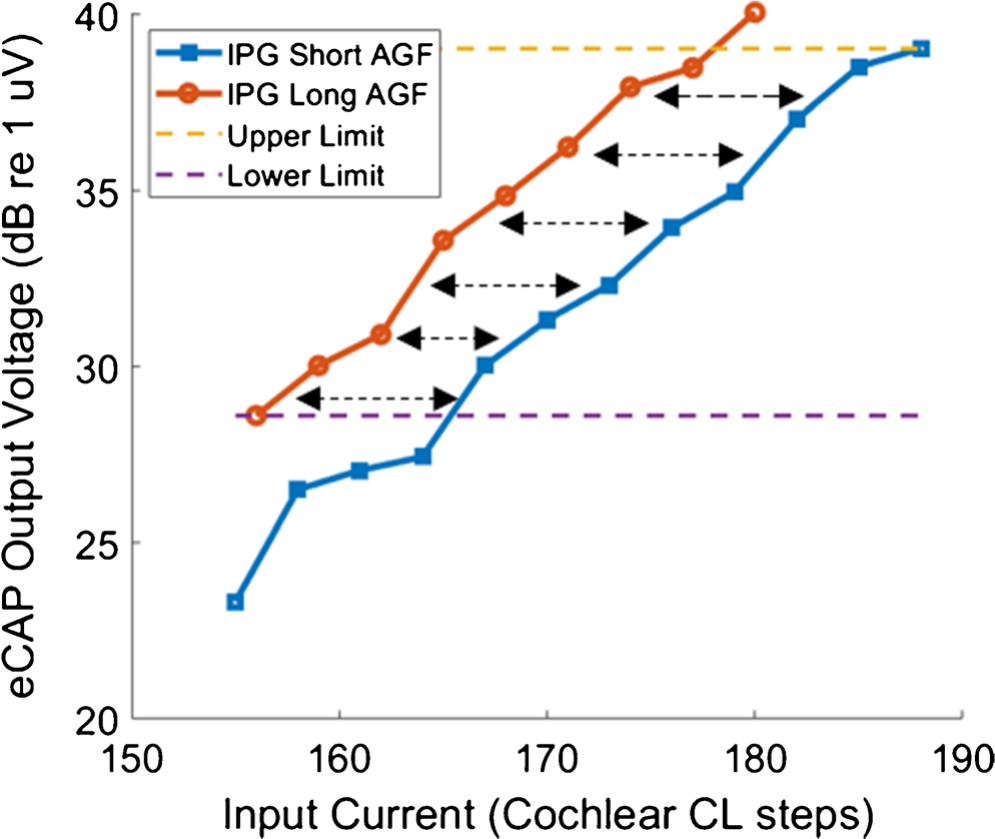
Amplitude growth functions (AGFs) for an example subject obtained at 8- and 40-μs IPG (blue and orange lines). The arrows illustrate the “IPG offset” between the two functions, which is defined as the mean difference between overlapping linear portions of the two ECAP AGFs (on a log input-log output scale)

We argue that a better method, which has been used by Kim et al. (2010) and by McKay and Smale (2017), is to measure the average dB offset between the linear portions of the ECAP AGFs for the different IPGs (Fig. 9). Each AGF was truncated to isolate linear portions of the AGFs that spanned the same ECAP output range. Because the output range is identical between conditions, the choice of linear or logarithmic scales for the output is not important. This method is better than the use of ECAP AGF slopes because it has been shown to correlate with SGN density in animal experiments (Prado-Guitierrez et al. 2006; Ramekers et al. 2014), and it isolates the effect of IPG from absolute measures such as absolute slope or *V*_*max*_ (provided that the input is in logarithmic units).

## Discussion

ECAPs are a valuable tool to assess the neural health of cochlear implant recipients. Absolute ECAP features may be affected by both neural and non-neural factors, such as electrode-modiolus distance, impedance, orientation of the electrodes relative to the neural population, and tissue growth around the electrode. In order to isolate neural factors from these non-neural factors, differential ECAP measures have been developed, where the difference (or ratio) between absolute ECAP features is measured on a particular electrode for different stimulus characteristics. Here we investigated analysis techniques for differential ECAP measures, showing that the IPG offset effect isolates neural health factors from non-neural factors, while the IPG slope effect does not. The article focusses on the effect of IPG, but the principle applies to any differential ECAP measure.

The re-analysis of the Prado-Guitierrez et al. (2006) animal data showed a significant correlation between the IPG offset effect and SGN density, consistent with similar data from Ramekers et al. (2014). The Prado-Guitierrez et al. (2006) data also showed a correlation between SGN density and the average dB shift between the ECAP AGFs at different IPGs, measured over a range of 20–80 % of the maximum ECAP output (*I*_*20–80%*_ offset), and it could be expected that the Ramekers et al. (2014) would show that correlation as well. There are a few advantages to calculating the IPG offset effect rather than the IPG slope effect. The first is that it has been correlated with SGN density in animals in two separate experiments with different pulse parameters. The second is that the fitting of the sigmoid or linear function is not as crucial to the calculation of the IPG offset effect as it is for the calculation of the IPG slope effect. When fitting a sigmoid or linear function for truncated human ECAP AGF data, some choices need to be made by the experimenter as to where the saturation level is (if using a sigmoid fit) or where the most linear portion of the AGF is (if using a linear fit).

The IPG offset effect should be interpreted as an estimate of neural health, rather than an estimate of neural survival. This distinction was demonstrated by our simple theoretical model (“Introduction” section) and can be supported by a recent study that measured the ECAP IPG offset effect in children diagnosed with cochlear nerve deficiency (CND) (Skidmore et al. 2020). CND is defined as a small or absent cochlear nerve, and CI outcomes for this pathology are generally poor. However, Skidmore et al. (2020) showed that the IPG offset effect was significantly larger for the CND group compared with the group of CI users without CND, consistent with neural *health* being better in the group with CND than the non-CND group, even though fewer neurons were present in the CND group. This result is consistent with temporal bone studies, which have shown that the remaining nerves in CND patients (while sparse) are not necessarily degenerated (Ylikoski and Savolainen 1984). Hence, compared with typical CI users with severe sensorineural hearing loss, the CND group might have a low number of relatively healthy nerves. The health of those nerves, and not the number, may be reflected by the significantly larger IPG offset effect in the CND group compared with the non-CND group.

Although we believe that the model and analyses presented here indicate that the IPG offset effect is dependent on neural health rather than on neural survival, they do not specify what aspect of neural health is responsible. Some insight into this issue comes from a recent study (Brochier et al. 2020), in which we measured the IPG offset from ECAP growth functions in 7 electrodes and for 10 participants. The IPG offset effect did not correlate, either across participants or electrodes, with two other proposed measures of neural health, namely, the effect of pulse rate and polarity on detection thresholds; this was true despite good test-retest reliability for each individual measure. We processed those stimuli using the biophysical model described by Joshi et al. (2017) and found that each measure could be modelled using a different component of the model. Reductions in the IPG offset effect could be implemented by modelling demyelination of the central axon, thereby increasing the membrane capacitance and reducing the membrane conductance. Importantly, it was not affected by the number of surviving neurons, consistent with the predictions of our model.

One thing to consider when measuring the IPG offset effect in truncated human data is that it is not entirely clear where the 50 % point in the AGF curve lies. Therefore, the method of McKay and Smale (2017) and Kim et al. (2010) should be used, where the average dB offset for overlapping linear regions is measured, rather than the dB offset for a single point. This calculation would still be consistent with the Prado-Guitierrez et al. (2006) measurement of the *I*_*20–80%*_ offset, which showed a correlation with SGN density. An IPG offset effect calculator MATLAB program has been added to our public git repository (https://github.com/tjbrochier/ECAP-AGF-Methods). The program will automatically calculate the IPG offset effect from ECAP AGFs in the format of three different implant companies (Cochlear, Advanced Bionics, and MedEl).

The theoretical model and the re-analysis of the Prado-Guitierrez et al. (2006) data suggest that the IPG slope effect is not a robust way to estimate neural health in humans. The only animal study that has shown a correlation between the IPG slope effect was performed by Ramekers et al. (2014). That study calculated the IPG effect on slope as the difference between linear slopes, so the measurement was prone to the influence of overall ECAP AGF slope. Specifically, increased SGN density may have simply increased the amplitude of the ECAP by a constant factor, which necessarily increased the linear slope, and with the linear difference in slope being larger for the longer IPG simply because those slopes were larger overall. It is also worth noting that this linear measure might not translate to accurate predictions of neural health or neural survival in humans. Absolute measures such as ECAP AGF slope and maximum ECAP amplitude are also good predictors of SGN density in guinea pigs (Shepherd and Javel 1997; Prado-Guitierrez et al. 2006; Ramekers et al. 2014). Compared with guinea pigs, humans have larger cochleas and longer durations of implantation, so the overall size of their ECAPs will be more greatly affected by electrode-modiolus distance and electrode orientation (both for the stimulating and recording electrodes) and by tissue growth around the electrode. All of these factors will affect the amplitude of the AGF and/or the range of stimulus levels used to measure the ECAP and, in a linear analysis, will affect the amount by which slope differs when the IPG is increased, even if the effect of the IPG is in fact a simple change in gain. Our re-analysis of the human ECAP AGF data from McKay and Smale (2017) showed that the (linear) IPG slope effect was correlated with the mean linear slope. If the mean linear slope were driven by non-neural factors, then so was the calculation of the IPG slope effect. The purpose of measuring the effect of IPG (or any differential ECAP measure) is to separate the neural factors from the non-neural factors, but this goal will not be achieved unless an appropriate analysis technique is used.

The analysis of similar data from Prado-Guitierrez et al. (2006), using either a difference between logarithmic slopes or a ratio between linear slopes, showed no correlation between SGN density and IPG slope effect. Furthermore, even when the IPG slope effect for the Prado-Guitierrez et al. (2006) data was calculated using the difference between linear slopes (as in Ramekers et al. (2014)), no correlation was found with SGN density. The Prado-Guitierrez et al. (2006) study did use longer phase durations than the Ramekers et al. (2014) study (100 μs versus 20–50 μs). The Fischer r to z transformation was used to compare the correlation coefficients between the IPG effect (calculated as the difference between linear slopes) and SGN density in the Prado-Guitierrez et al. (2006) and Ramekers et al. (2014) data. The Prado-Guitierrez et al. (2006) correlation coefficient was significantly smaller than the Ramekers et al. (2014) correlation coefficient for the phase duration of 20 μs, but not for the phase duration of 50 μs. Either the correlation between SGN density and IPG slope effect was not robust to the longer phase durations used in the Prado-Guitierrez et al. (2006) study or the correlation in the Ramekers et al. (2014) data was driven largely by steeper ECAP AGF slopes in the NH implanted group. In order to settle the question of whether the IPG slope effect is correlated with SGN density, the Ramekers et al. (2014) data should be re-analysed using the ratio between linear slopes, rather than the difference.

Despite our demonstration that the IPG slope effect does not isolate the neural health factor, there is evidence in the literature that it does correlate with speech perception. Schvartz-Leyzac and Pfingst (2018) measured the IPG slope effect and speech perception in the left and right ears of bilateral CI users. They calculated the IPG slope effect as the difference between linear slopes, in a method similar to Ramekers et al. (2014). A correlation was found between the between-ear difference in mean IPG slope effect and speech reception threshold (*r* = − 0.84, *p* = 0.002) and consonant identification (*r* = 0.75, *p* = 0.006). As noted in our simple model, the IPG slope effect measured in this way will depend on *r*, *s*, and *n*, and although *r* should not affect speech perception, the observed correlation may have been due to *s*, *n*, or both*.* It is also important to note that Schvartz-Leyzac and Pfingst (2018) averaged the IPG slope effects across all electrodes in each ear. By averaging across electrodes, the between-subject effects of different recording and stimulating electrode characteristics (impedance, EMD, orientation) are probably reduced and the average IPG slope effect might then primarily reflect contributions from overall neural health or neural survival. However, if the IPG slope effect was being used to identify poor electrodes within a single subject, the effect of recording electrode and stimulation electrode would be stronger, perhaps even larger than the effect of overall neural health. Looking at the effect of EMD, for example, data from Long et al. (2014) shows that the mean EMD across the electrode array for 10 subjects was approximately 0.9 mm, with a standard deviation of only 0.17 mm. The mean standard deviation of EMD across the array for each individual subject, however, was 0.36 mm. Therefore, the influence of EMD on the calculation of the IPG slope effect would be stronger for a within-subject comparison of different electrodes than it would for a between-subject comparison of average IPG slope effect.

## Conclusion

The use of the IPG effect on ECAP AGFs as an estimate of neural health was evaluated using a simple theoretical model, guinea pig ECAP AGFs, and human ECAP AGFs. Both the theoretical model and the re-analysis of the animal data suggest that the IPG slope effect, measured using either a linear or logarithmic input-output scale, is not a robust estimate of neural health. The best estimation of neural health, based on its correlation with SGN density in two separate animal studies, is the IPG offset effect (using dB current on the x-axis) for the two different IPG conditions. This metric is independent from absolute ECAP measures such as slope and maximum amplitude, which can be influenced by non-neural factors.
